# Elucidation of the Specific Formation of Homo- and Heterodimeric Forms of ThbZIP1 and Its Role in Stress

**DOI:** 10.3390/ijms150610005

**Published:** 2014-06-04

**Authors:** Xianguang Nie, Xiaoyu Ji, Yujia Liu, Lei Zheng, Yucheng Wang

**Affiliations:** 1 State Key Laboratory of Tree Genetics and Breeding (Northeast Forestry University), 26 Hexing Road, Harbin 150040, China; E-Mails: nxg_ok@126.com (X.N.); zhenglei8123@126.com (L.Z.); 2 Key Laboratory of Biogeography and Bioresource in Arid Land, Xinjiang Institute of Ecology and Geography, Chinese Academy of Sciences, Urumqi 830011, China; E-Mail: jixy0219@163.com; 3 College of Food Engineering, Harbin University of Commerce, 1 Xuehai Street, Harbin 150028, China; E-Mail: lyjizbd@126.com

**Keywords:** bZIP (basic leucine zipper proteins) transcription factors, heterodimerization, protein–protein interaction, two-hybrid analysis, *Tamarix hispida*

## Abstract

Protein–protein interactions are important for the molecular understanding of the biological processes of proteins. The dimerization of bZIPs (basic leucine zipper proteins) is involved in modifying binding site specificities, altering dimer stability, and permitting a new set of specific protein-to-protein interactions to occur at the promoter. In the present study, we studied the whether ThbZIP1 form homo- and heterodimers using the yeast two-hybrid method. Five *bZIP* genes were cloned from *Tamarix hispida* to investigate their interaction with ThbZIP1. Our results showed that ThbZIP1 can form homodimers with itself, and three out of five bZIPs could interact with the ThbZIP1 protein to form heterodimers. Real-time RT-PCR results suggested that these *ThbZIPs* can all respond to abiotic stresses and abscisic acid (ABA), and shared very similar expression patterns in response to NaCl, ABA or PEG6000. Subcellular localization studies showed that all *ThbZIPs* are targeted to the nucleus. Our results showed that ThbZIP1 are dimeric proteins, which can form homo- or heterodimers.

## 1. Introduction

Basic leucine zipper proteins (bZIPs) are a large family of transcription factors (TFs) in plants, bZIP TFs contain a characteristic and highly conserved basic domain with two structural features: the basic/positively charged sequence, which serves as both a DNA binding and nuclear localization element, and a leucine zipper dimerization element [1,2]. In the model plant *Arabidopsis thaliana*, 75 bZIP proteins have been identified and classified into 10 groups based on their sequence similarity [3]. In general, bZIP TFs bind to DNA as homo- or heterodimers [4], and the tendency of specific bZIP TFs to form homo- or heterodimers depends on the amino acid sequences in their Leu zippers. Consequently, dimer formation enables the establishment of complex regulatory networks based on combinatorial interactions.

Protein–protein interactions have been increasingly considered as important for the molecular understanding of biological processes [5]. In particular, yeast two-hybrid approaches have been very successful in identifying putative protein interaction partners [6]. Heterodimerization in yeast has been confirmed *in vitro* by EMSA (electrophoretic mobility shift assay) studies and in plants using a protoplast two-hybrid system [7]. Biochemical approaches, such as coimmunoprecipitation (Co-IP), or cytological methods, such as fluorescence resonance energy transfer (FRET) [8] or bimolecular fluorescence complementation (BiFC) [9,10,11], have been used. 

In general, the heterodimerization of bZIP has been little addressed in plants. TFs of the bZIP family bind to DNA predominantly as homo- or heterodimers [4,12,13]. The yeast two-hybrid system assay offers a valuable tool for analyzing heterodimerization in plants [7]. The formation of bZIP homo- or heterodimers offers huge combinatorial flexibility to regulatory transcription systems. Via heterodimerization, DNA-binding specificity and affinity, transactivation properties and overall cell physiology might be altered [14]. In plants, three tobacco bZIP heterodimerization partners have been isolated which form specific heterodimers with BZI-1, namely BZI-2, BZI-3, and BZI-4 (bZIP TFs) [15]. In particular, members of the C/S network have been implicated in the stress response [16], amino acid metabolism [17,18], and energy homeostasis [19,20]. A publication deals with the rice bZIP factor LIP19/OBF1 as being implicated in the regulation of low-temperature-induced genes [21]. We also found that AtbZIP28 forms a transcriptional complex with a NF-YA4/NF-YB3/NF-YC2 trimer, and AtbZIP28 heterodimerizes most efficiently with AtbZIP17 and AtbZIP60. As the BZI family factors show a preference to form heterodimers [15], heterodimerization between these groups is strongly preferred in comparison to homotypic dimerization [22]. AtbZIP10 and AtbZIP25 physically interact with ABI3, which in turn enhances the *in vitro* DNA binding of bZIP proteins to SSP (seed storage proteins) promoters as well as their *in vivo* activation capacity [23]. AtbZIP53 plays a pivotal and crucial role in the quantitative control of *MAT* gene transcription levels by cooperating with several TFs to form enhanceosome-like protein complexes [24]. AtbZIP53 and the group-C heterodimerization partner AtbZIP10 were found to colocalize; they control ACTCAT-mediated ProDH transcription in a synergistic manner. Furthermore, they provide *in vivo* evidence that ProDH is not regulated by a single bZIP, but by a complex heterodimerization network of group-S1/C bZIP factors [17].

*In vivo* protein–protein interactions are frequently studied with a yeast two-hybrid analysis. However, interactions detected in yeast might differ considerably in plant systems. The efficiency of the system was tested by examining the homo- and heterodimerization properties of basic leucine zipper (bZIP) transcription factors. *Tamarix hispida* Willd. is a woody halophyte that can grow well in drought prone soils and soils with high levels of salinity. Previously, we had cloned six *bZIP* genes (*ThbZIP1*, *ThbZIP2*, *ThbZIP4*, *ThbZIP5*, *ThbZIP6*, and *ThbZIP7*) from eight transcriptomes of *T. hispida* roots treated with NaHCO_3_ for 0, 12, 24, or 48 h [25]. The transgenic tobacco overexpression of *ThbZIP1* showed improved salt tolerance [26]. Overexpression of *ThbZIP1* led to a reduction in the cellular levels of ROS (reactive oxygen species), cell death and water loss under salt, drought, and ABA treatment conditions [27]. In this work, we further demonstrated that the bZIP factors are dimeric proteins. We also investigated whether ThbZIP1 could homo- or heterodimerize by the yeast two-hybrid system. 

## 2. Results and Discussion

### 2.1. Analysis of the Hetero- and Homodimers of ThbZIP1

The function of *ThbZIP1* (GenBank number FJ752700) had been studied previously. The expression of ThbZIP1 is induced by ABA, salt and drought. ThbZIP1 is found to specifically bind to ACGT elements, with the highest binding affinity to the C-box, followed by the G-box and lastly the A-box. The Arabidopsis plant that overexpressed ThbZIP1 had an increased tolerance to drought and salt compared with wild-type (Col-0) Arabidopsis. In addition, ROS levels, cell death and water loss rates in transgenic plants were significantly reduced compared with WT plants under salt and drought stress conditions. Microarray analyses showed that many ROS scavenging genes were upregulated by ThbZIP1 to enhance ROS scavenging ability under salt stress conditions [27]. The bZIP TFs are dimeric proteins, and can homo- and heterodimerize via a coiled-coil structure [4]. The dimerization of bZIPs contributes to cellular regulation by modifying binding site specificities, altering dimer stability, and permitting a new set of specific protein-to-protein interactions to occur at the promoter [28]. In the present study, we further studied whether ThbZIP1 could homo- or heterodimerize. Besides ThbZIP1, five *bZIP* genes (*ThbZIP2*, *ThbZIP4*, *ThbZIP5*, *ThbZIP6*, and *ThbZIP7*) with full ORFs (open reading frames) were also cloned from *T. hispida* by transcriptome analyses. The phylogenetic tree showed that these six ThbZIPs and bZIP proteins from different plants formed three main subgroups: subgroup 1 contained ThZIP1, ThZIP6, and ThZIP7; subgroup 2 contained ThZIP4 and ThZIP5; and subgroup 3 contained ThZIP2 only. ThbZIP1 was most similar with AtbZIP53 from *A. thaliana* (Figure S1)*.*

**Figure 1:**
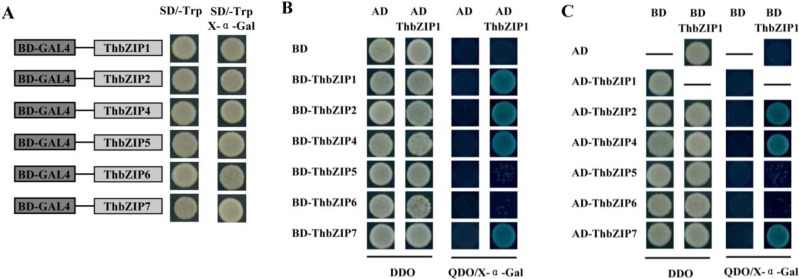
Autoactivation, homodimeric, and heterodimeric analysis of ThbZIPs using a yeast two-hybrid assay (Y2H). (**A**) Transactivation assay of ThbZIPs. Fusion proteins of the GAL4 DNA-binding domain and ThbZIPs were checked for their transactivation activity using the Y2H system. None of the ThbZIPs had autoactivation or toxicity, and were therefore competent for a Y2H analysis; (**B**) ThbZIP1 was cloned into a pGBKT7 vector (Clontech) and interacted respectively with itself and the other five bZIP proteins that were cloned into pGADT7 (prey); and (**C**) ThbZIP1 was cloned into pGADT7 vector (prey) and interacted with itself as well as the other five bZIP proteins cloned into the pGBKT7 vector (baits). The yeast cells were grown on SD/-Trp-Leu (DDO) and selective dropout media: SD/-Ade-Trp-Leu/-His /X-α-Gal (QDO/X-α-Gal).

The ORFs of *ThbZIP1*, *ThbZIP2*, *ThbZIP4*, *ThbZIP5*, *ThbZIP6*, and *ThbZIP7* were respectively cloned into pGBKT7 (Clontech, Palo Alto, CA, USA) and their autoactivation or toxicity were analyzed. Our results showed that all of these bZIP proteins had no autoactivation or toxicity in their single form (Figure 1A), and were therefore suitable for a yeast two-hybrid analysis to test for homodimerization, in which ThbZIP1 served as both bait and prey. The results showed that ThbZIP1 could interact with itself (Figure 1B), which indicated that it can form a homodimer. 

To analyze the heterodimers of ThbZIP1, it served as bait to interact with other ThbZIPs. The results showed that ThbZIP2, 4 and 7 could interact with ThbZIP1, but ThbZIP5 and ThbZIP6 could not interact with ThbZIP1 (Figure 1B). To further test these heterodimerizations, all of the bZIP factors beside ThbZIP1 were used as the bait, and respectively interacted with ThbZIP1 as the prey. The results consistently showed that ThbZIP2, ThbZIP4, and ThbZIP7 interacted with ThbZIP1, but ThbZIP5 and ThbZIP6 failed to interact with ThbZIP1. Taken together, these results suggested that ThbZIP1 can form homodimers, as well as heterodimers, with ThbZIP2, ThbZIP4, and ThbZIP7 (Figure 1C). 

### 2.2. The Binding of ThbZIP2, ThbZIP4, and ThbZIP7 to C-Box Motifs

**Figure 2:**
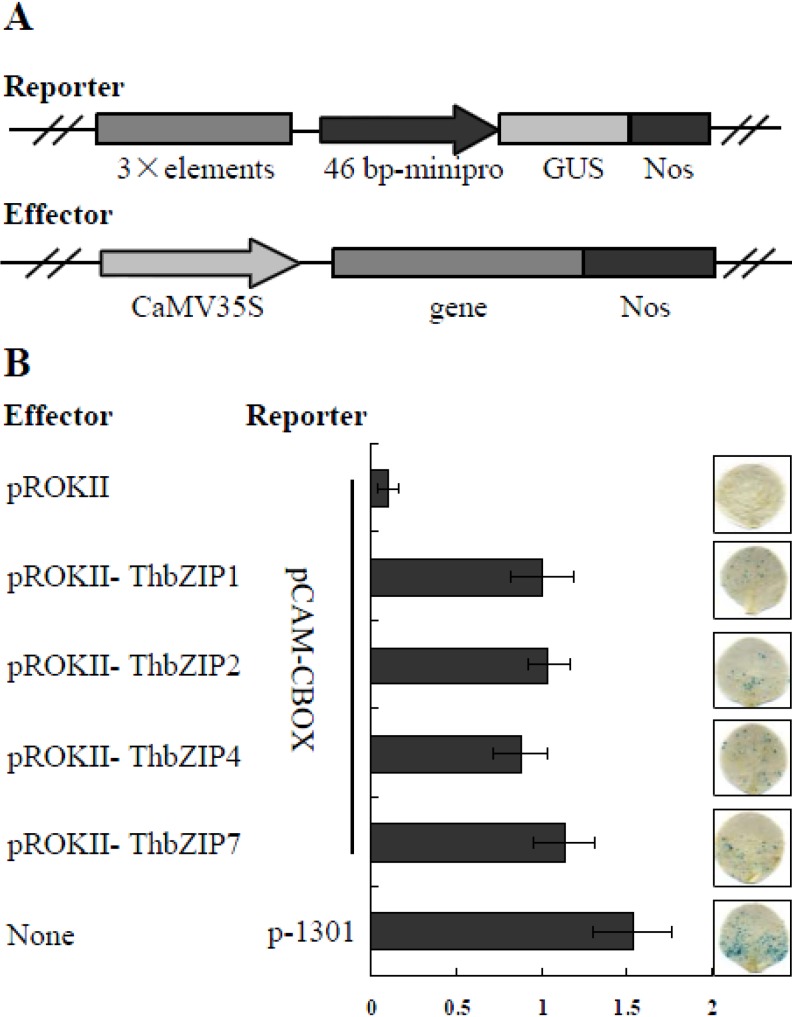
Transactivation potential of selected ThbZIP transcription factors. (**A**) To further verify these interactions, the three tandem copies of the C-box were fused to the minimal 35S promoter (−46 to +1) and the resultant fusion was cloned into pCAMBIA1301 to substitute its 35S promoter driving GUS (β-glucuronidase) (constructs containing the C-box were named pCAM-C). The effector vector was constructed by cloning the full ORFs (open reading frames) of ThbZIP genes into pROKII under the drive of the 35S promoter (pROKII-*ThbZIPs*) (see Table S1 for the primers used). Both the reporter and effector vectors were co-transformed into tobacco leaves using particle bombardment; (**B**) GUS staining and GUS activity assay were determined as described above.

A previous study had shown that the ThbZIP1 can specifically bind to C-box motifs, and we therefore aimed to determine whether this was the case for ThbZIP2, ThbZIP4, and ThbZIP7. A quantitative assay for protein–DNA interactions was performed *in planta* using a transient expression system. A reporter vector (pCAM-C-box) was constructed using three tandem copies of the C-box fused to the minimal 35S promoter to drive GUS (β-glucuronidase). The effector vectors were constructed by cloning the full ORFs of *ThbZIP2* (pROKII-ThbZIP2), *ThbZIP4* (pROKII-ThbZIP4), and *ThbZIP7* (pROKII-ThbZIP7), respectively, into pROKII under the control of the 35S promoter (Figure 2A). The transformation of each effector into tobacco leaves together with the reporter vector was performed using particle bombardment. GUS activity was determined to study the binding affinity of ThbZIP2, ThbZIP4, and ThbZIP7 to the C-box motif. As shown in Figure 2B, the transformation of ThbZIP2, ThbZIP4, or ThbZIP7 results in high levels of GUS activity. These results suggested that all three proteins can interact with the C-box motif.

### 2.3. The Expression of ThbZIPs in T. hispida Can Be Induced by Salt, ABA (Abscisic Acid), and Drought

**Figure 3:**
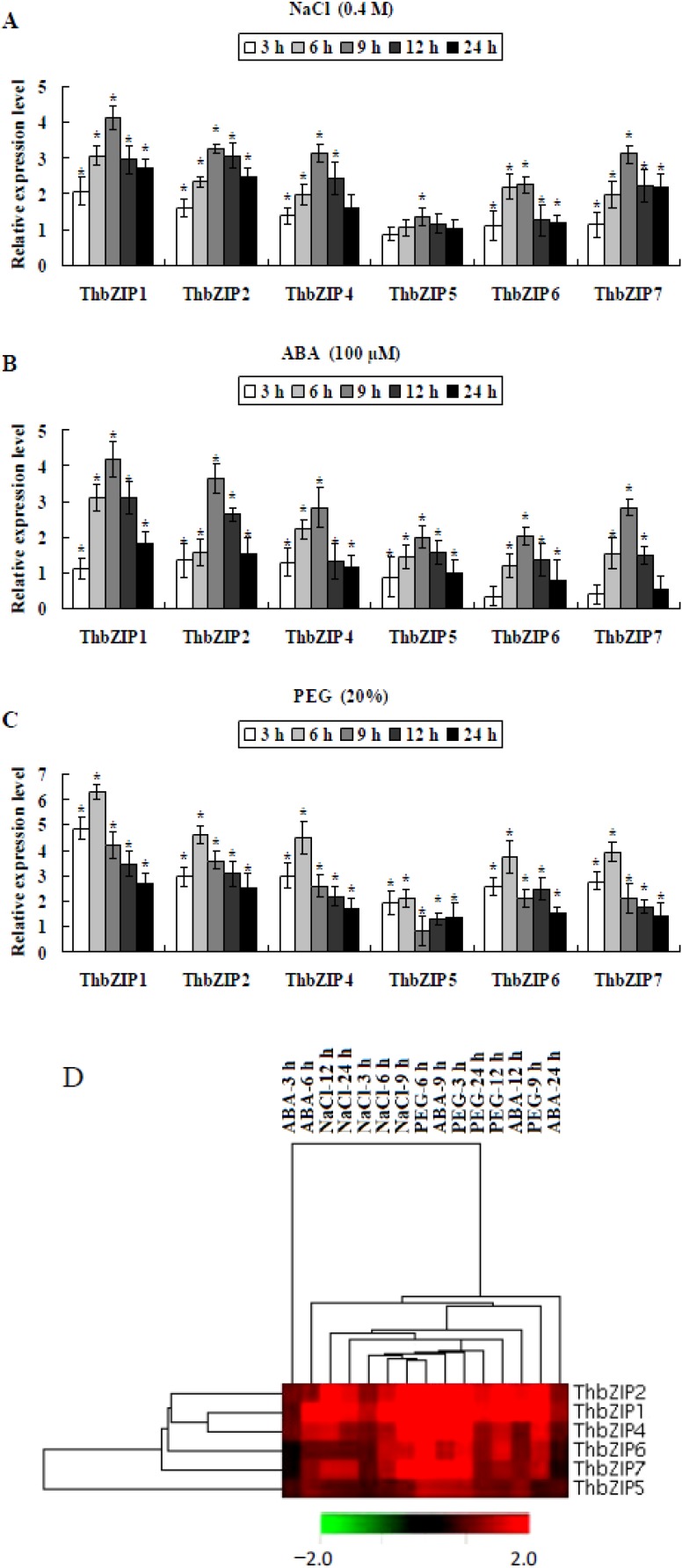
The expression patterns of ThbZIPs in response to abiotic stresses and ABA (abscisic acid). (**A**,**B**,**C**) The expression patterns of ThbZIPs in response to salt, ABA, and osmotic stress treatments. The error bars were obtained from multiple replicates of the real-time PCR assays. Data are shown as the means ± SD from three independent experiments. * Significant (*t*-test, *p* < 0.05) differences compared with the expression at 0 h; and (**D**) Hierarchical cluster analysis of the expression of ThbZIPs in response to abiotic stress. All the ratios were log2-transformed for cluster analysis. Log ratios of 0 (ratios of 1) are colored black, and induction or repression ratios are colored red or green with increasing intensity, respectively.

As ThbZIP1 can interact with ThbZIP2, ThbZIP4, and ThbZIP7, we further compared changes in their expression level in response to treatments with salt (Figure 3A), drought (Figure 3B), and ABA (Figure 3C). The results showed that the expression patterns of all the studied *ThbZIPs* were relatively similar when exposed to all three treatments (Figure 3D). Expression levels rose significantly and reached their peak level at 9 h for salt and ABA treatments, but reached peak level at 6 h after PEG-induced osmotic stress. As ThbZIP2, ThbZIP4, and ThbZIP7 can dimerize with ThbZIP1, and also are significantly induced by NaCl, PEG, or ABA treatments, these findings suggest that they may also be involved in the abiotic stress response along with ThbZIP1. A hierarchical cluster analysis was performed, and the results showed that ThbZIP1 shared similar expression patterns in response to salt, drought, and ABA treatments. Besides ThbZIP5, all of the ThbZIP genes showed similar expression patterns (Figure 3E). All of these genes reached their peak expression level after NaCl stress at 6 or 9 h, PEG stress at 6 h, and ABA treatment at 9 h (Figure 3E). Taken together, these results suggested that ThbZIP1 may share the same regulatory pathway in response to abiotic stress with ThbZIP2, ThbZIP4, or ThbZIP7, and may play roles in stress tolerance by dimerizing with ThbZIP2, ThbZIP4, or ThbZIP7.

### 2.4. Targeting ThbZIPs to the Nucleus

**Figure 4:**
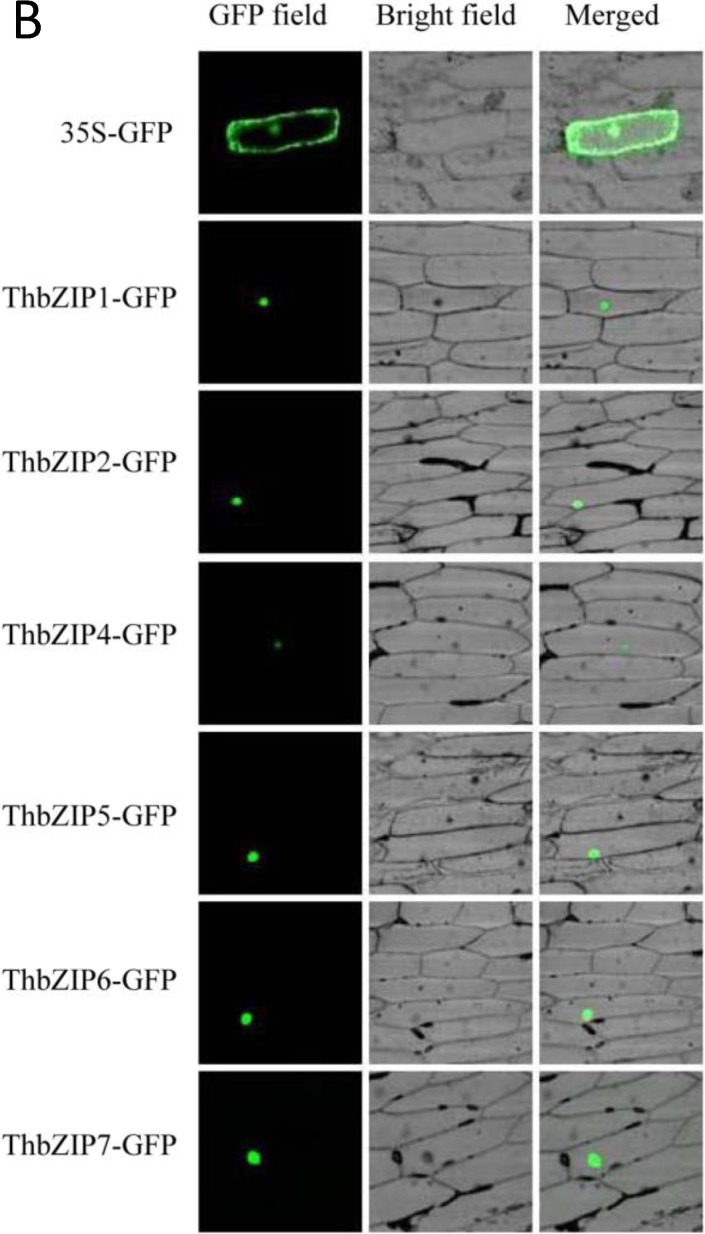
The subcellular localization of ThbZIPs. (**A**) Schematic map of the 35S-GFP control constructs and the fusion construct for the *ThbZIPs*-*GFP* vector; (**B**) The fusion construct for *ThbZIPs*-*GFP* and the 35S-GFP control constructs were introduced into the onion epidermal cells by particle bombardment.

The subcellular localization patterns of *ThbZIPs* were determined using the *ThbZIPs-GFP* fusion gene under the control of the CaMV 35S promoter. GFP driven by the 35S CAMV promoter was used as the control. The scheme of these constructs is shown in Figure 4A. The *ThbZIPs-GFP* fusion gene and the *GFP* control were transformed into onion epidermal cells by particle bombardment. The green fluorescent signals of *ThbZIPs*-*GFP* were localized to the nucleus of onion epidermal cells, whereas GFP was found to be uniformly distributed throughout the cells (Figure 4B). These results indicated that the studied ThbZIPs are all nuclear proteins.

## 3. Experimental Section

### 3.1. Plant Materials and Growth Conditions

Seedlings of *T. hispida* were planted in pots that contained a mixture of turf peat and sand (2:1 *v*/*v*) in a greenhouse under controlled conditions of 70%–75% relative humidity, light/dark cycles of 14/10 h, maintained at 24 °C. To induce abiotic stresses, the 2-month-old seedlings were watered on their roots with a solution of 0.4 M NaCl, 100 μM ABA, and 20% (*w/v*) PEG6000 for 0, 3, 6, 9, 12, and 24 h. After each stress treatment, at least 20 seedlings were harvested and pooled for further study.

### 3.2. Cloning the ThbZIP Gene Sequences

Previously, eight transcriptomes were built from roots of *T. hispida* treated with NaHCO_3_ for 0, 12, 24, and 48 h (two biological replicates were set at each time point). In total, 47,324 unigenes were generated after the de novo assembly of these transcriptomes using SOAPdenovo [25]. After sequence assembly and unigene functional annotation, six unique ThbZIPs with full ORFs were obtained: *ThbZIP1* (FJ752700), *ThbZIP2* (JX169811), *ThbZIP4* (JX169812), *ThbZIP5* (JX169813), *ThbZIP6* (JX169814), and *ThbZIP7* (JX169815). In total, six unique bZIP genes were identified from the transcriptomes of *T. hispida*. The amino acid sequences of ThbZIPs and 31 Arabidopsis bZIP genes were aligned, and an unrooted NJ (neighbor-joining) tree was constructed using MEGA 6.06 [29]. The sequences of the Arabidopsis bZIP domain proteins were downloaded from the Arabidopsis genome TAIR 9.0 [30].

### 3.3. Yeast Transactivation Activity Analysis

The yeast strain Y2H harboring the *LacZ* and *HIS*3 reporter genes were used as an assay system (Clontech). The full length or partial fragments coding sequences of *ThbZIPs* (*ThbZIP1-2*, *ThbZIP4-7*) were cloned into the pGBKT7 vector, containing the GAL4 DNA binding domain, according to the protocol provided by the manufacturer (Clontech). All constructs were transformed into the yeast strain Y2H containing the *LacZ* and *HIS*3 reporter genes. The transformed colonies were confirmed by PCR and were then inoculated by growth for 3–5 days on SD/–Trp and SD/–Ade/–Trp/–Leu medium, including the LacZ filter-lift assay with 5-bromo-4-chloro-3-indoxyl-α-d-galactopyranoside (X-α-Gal), to eliminate residual expression of the leaky HIS3 reporter gene. The transcription activation activities were evaluated according to their growth status.

### 3.4. Autoactivation, Heterodimer, and Homodimer Assays for ThbZIP1

PCR fragments of ThbZIPs without the terminal codon was inserted into the sites of the pGBKT7 vector (Clontech), containing *Bam*HI and *Eco*RI sites, as bait. Six *ThbZIP* genes were cloned with full ORFs from *T. hispida*, and the ThbZIPs were cloned into pGADT7 as the prey constructs (see Table S1 for a list of primers used). The constructs were confirmed by DNA sequencing. The pGBKT7 vector containing the GAL4 DNA binding domain and the pGADT7-Rec2 vector were described by the Matchmaker™ Gold Yeast Two-Hybrid System (Clontech). The PCR products were inserted into vectors using an In-Fusion™ Advantage PCR Cloning Kit (Clontech).

The interaction of *ThbZIP1* with itself and the other bZIPs were investigated in yeast with a two-hybrid system. The bZIPs that were able to interact with ThbZIP1 (positive clones) were cloned into pGBKT7 and transformed into the Y2H Gold yeast strain to test their autoactivation and toxicity. *ThbZIP1* was cloned into pGADT7-Rec2 as a prey and allowed to interact with the baits to further test their interactions.

### 3.5. Transient Expression Assays

To further verify these interactions, the three tandem copies of the C-box were respectively fused to the minimal 35S promoter (−46 to +1) to drive GUS, and designed as pCAM-C-box vectors (see Table S2 for primers used). The effector vectors were constructed by cloning the full ORF of ThbZIPs genes into pROKII driven by the 35S promoter (named pROKII-ThbZIP1, pROKII-ThbZIP2, pROKII-ThbZIP4, pROKII-ThbZIP5, pROKII-ThbZIP6, and pROKII-ThbZIP7). Both of the reporter vectors and effector vectors were co-transformed into tobacco leaves using the particle bombardment method (Bio-Rad, Hercules, CA, USA), with combinations of ThbZIPs at different ratios. A GUS histochemical staining assay was performed as described by Jefferson [31], and the GUS activity levels were determined according to the previously described method [32]. 

### 3.6. Real-Time PCR Analysis of Gene Expression

Total RNA of each sample was extracted using the CTAB method [31], and was then treated with DNaseI (Promega, Madison, WI, USA) to remove any residual DNA. RNA concentration was measured using a BioPhotometer plus (Eppendorf, Hamburg, Germany). Approximately 1 μg of total RNA was reverse-transcribed to cDNA using 1 μM of oligodeoxythymidine primer. The synthesized cDNA was diluted to 100 μL with sterile water and used as template for real-time RT-PCR.

The real-time RT-PCR was performed using *Actin* (FJ618517), *α-tubulin* (FJ618518), and *β-tubulin* (FJ618519) as internal controls (see Table S3 for primers used). PCR was performed on a MJ Research OpticonTM2 instrument with the following conditions: 94 °C for 30 s, 45 cycles of 94 °C for 12 s, 58 °C for 30 s, 72 °C for 40 s, and 80 °C for 1 s for a plate reading. The relative expression levels of the products were calculated according to the 2^−ΔΔ*C*t^ method [33]. Relative gene expression level was calculated as the transcription level under stress treatment divided by the transcription level of the controls (*i.e.*, samples from plants grown under normal conditions and harvested at the same treatment time points). Hierarchical cluster analysis was performed using Cluster 3.0 software [34]. The settings for the calculations were that similarity was measured by standard correlation and the clustering method used was average linkage. The genes with similar profiles were clustered together as a taxonomic tree.

### 3.7. The Subcellular Localization of the ThbZIP Proteins

The coding region of ThbZIPs were ligated in-frame to the *N*-terminal of the green fluorescent protein (GFP) to generate the *ThbZIPs*-*GFP* fusion gene under the control of the CaMV 35S promoter (*ThbZIPs*-*GFP*) (see Table S4 for primers used), and the GFP driven by the CaMV 35S promoter (35S-GFP) was used as a control. The plasmid encoding the *ThbZIPs*-*GFP* fusion protein and the GFP were introduced into onion epidermal cells by particle bombardment (Bio-Rad). The transformed cells were analyzed using confocal laser scanning microscopy on an LSM710 microscope (Zeiss, Jena, Germany).

### 3.8. Statistical Analysis

Data analyses were performed using SPSS 16.0 (SPSS Inc., Chicago, IL, USA) software. For all the analyses, the significance level was set at *p* < 0.05. Sample variability is given as the standard deviation (SD) of the mean.

## 4. Conclusions

The current data reveal that ThbZIP1 can form homodimers with itself, and three out of five bZIPs could interact with the ThbZIP1 protein to form heterodimers. Real-time RT-PCR results suggested that these ThbZIPs could all respond to abiotic stresses and ABA, and shared very similar expression patterns in response to NaCl, ABA, or PEG6000. A subcellular localization study showed that these ThbZIPs were all targeted to the nucleus. Our results showed that ThbZIP1 is a dimeric protein, which could form homo- or heterodimers. In addition, the bZIPs studied in this report may play important roles in abiotic stress tolerance. Drought and salinity are the most common adverse environments encountered by plants, and are the main factors that restrict the yield of crop plants. Therefore, breeding transgenic plants with improved abiotic stress tolerance is an important goal in agricultural production. These bZIP genes from *Tamarix hispida* may have great potential for the creation of transgenic crop plants to improve salt and drought tolerance by genetic engineering methods, which will increase yields in adverse environments to meet the food demands by increasing populations.

## Supplementary Files

Supplementary File 1 Supplementary Information (PDF, 133 KB)
