# Supplementary material for: Elucidation of the Specific Formation of Homo- and Heterodimeric Forms of ThbZIP1 and Its Role in Stress
Source: Int J Mol Sci. 2014 Jun 4;15(6):10005–17. doi: 10.3390/ijms150610005 (PMC4100136; doi:10.3390/ijms150610005)
Supplement: Supplementary File 1 — Supplementary Information (PDF, 133 KB) [file ijms-15-10005-s001.pdf]

Supplementary Information

Figure S1. Phylogenetic analysis of bZIP protein sequences. Phylogenetic relationship of bZIP proteins from *T. hispida* and Arabidopsis. The amino acid sequences of ThbZIPs and 31 Arabidopsis bZIP genes were aligned, and the unrooted NJ tree was constructed using MEGA 6.06. The sequences of the Arabidopsis bZIP domain proteins were downloaded from the Arabidopsis genome TAIR 9.0 [1].

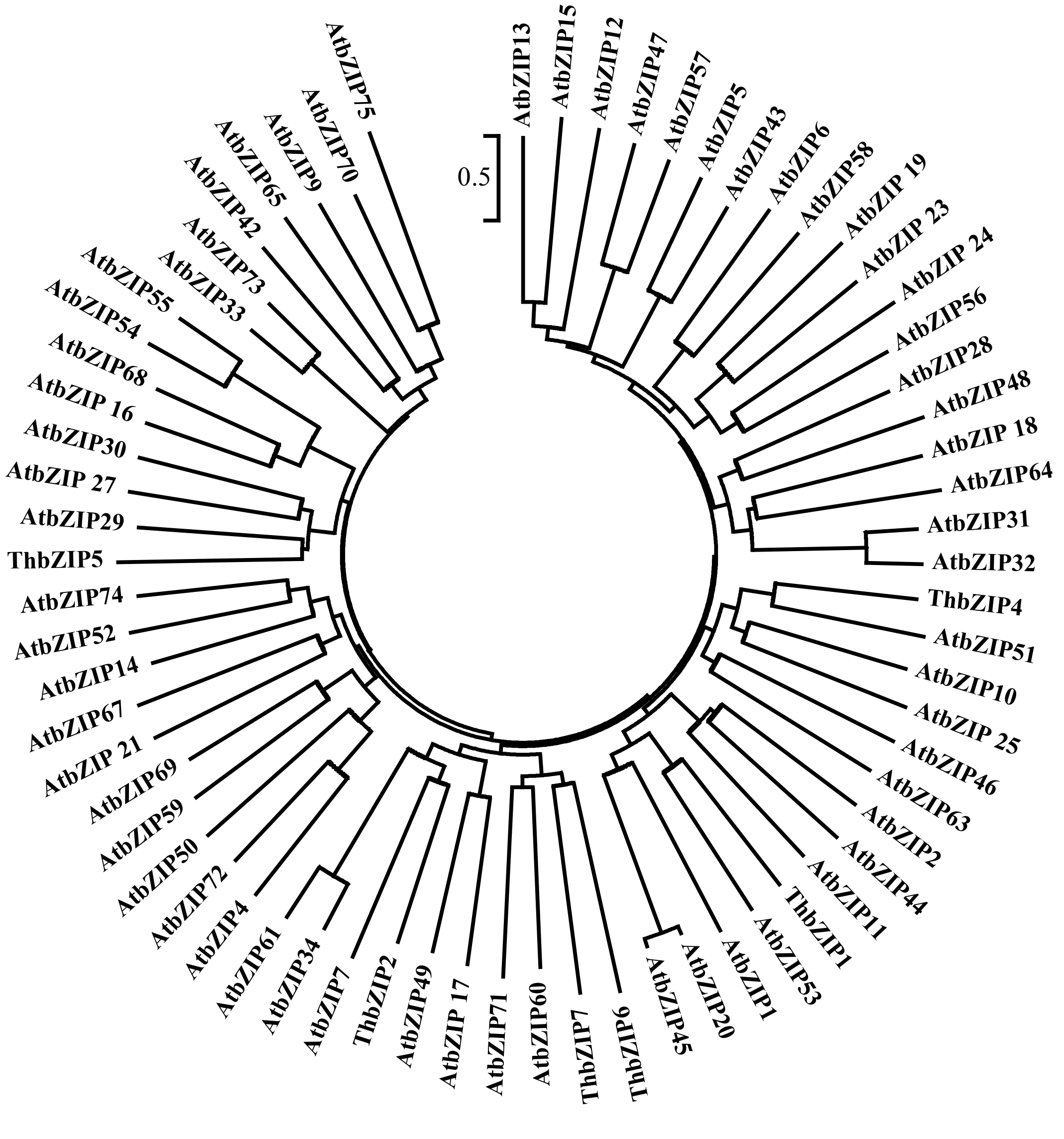

Table S1. The primers used for the yeast two-hybrid system analysis.

Primer Names	Primers Sequences (5'–3')
AD-ThbZIP1F	AAGCAGTGGTATCAACGCAGAGTGGCCATTATG GCCCATGTATCAACCCGTGAGTTCTGGT
AD-ThbZIP1R	TCTAGAGGCCGAGGCGGCCGACATGTTAGAACTGAAACATATCAGCGGT
AD-ThbZIP2F	AAGCAGTGGTATCAACGCAGAGTGGCCATTAT GGCCCATGGGGTCTTCGAACTCGAAGCCA
AD-ThbZIP2R	TCTAGAGGCCGAGGCGGCCGACATGTCAGAGCCTTCTTCTACCATTTGT
AD-ThbZIP4F	AAGCAGTGGTATCAACGCAGAGTGGCCATTAT GGCCCATGAACCCAAATTACTACTCCAAG
AD-ThbZIP4R	TCTAGAGGCCGAGGCGGCCGACATGTTATTGAATCCTATGATTATAGTC
AD-ThbZIP5F	AAGCAGTGGTATCAACGCAGAGTGGCCATTATG GCCCATGTTGTCCACAATTCCGGCTAAT
AD-ThbZIP5R	TCTAGAGGCCGAGGCGGCCGACATGTTATGGCCATGCAAAAGACTGCTC
AD-ThbZIP6F	AAGCAGTGGTATCAACGCAGAGTGGCCATTAT GGCCCATGGCTTGTATGAAGTTAGGATCT
AD-ThbZIP6R	TCTAGAGGCCGAGGCGGCCGACATGTCACAGGTTTTTCTTGTGCCTTGG
AD-ThbZIP7F	AAGCAGTGGTATCAACGCAGAGTGGCCATTAT GGCCCATGGCGATGCAGACTGGAGTAGGG
AD-ThbZIP7R	TCTAGAGGCCGAGGCGGCCGACATGCTACAATACTAAACCCGCTGGCTT
BD-ThbZIP1F	CATGGAGGCCGAATTCATGTATCAACCCGTGAGTTCTGGT
BD-ThbZIP1R	GCAGGTCGACGGATCCGAACTGAAACATATCAGCGGTATT
BD-ThbZIP2F	CATGGAGGCCGAATTCATGGGGTCTTCGAACTCGAAGCCA
BD-ThbZIP2R	GCAGGTCGACGGATCCTTACGAGCCTTCTTCTACCATTTGT
BD-ThbZIP4F	CATGGAGGCCGAATTCATGAACCCAAATTACTACTCCAAG
BD-ThbZIP4R	GCAGGTCGACGGATCCTTGAATCCTATGATTATAGTCTAG
BD-ThbZIP5F	CATGGAGGCCGAATTCATGTTGTCCACAATTCCGGCTAAT
BD-ThbZIP5R	GCAGGTCGACGGATCCTGGCCATGCAAAAGACTGCTCATG
BD-ThbZIP6F	CATGGAGGCCGAATTCATGGCTTGTATGAAGTTAGGATCT
BD-ThbZIP6R	GCAGGTCGACGGATCCCAGGTTTTTCTTGTGCCTTGGATG
BD-ThbZIP7F	CATGGAGGCCGAATTCATGGCGATGCAGACTGGAGTAGGG
BD-ThbZIP7R	GCAGGTCGACGGATCCCAATACTAAACCCGCTGGCTTTAA

Table S2. Primers used for construction of the reporter and effector vectors.

Primer Names	Primers Sequences (5'–3')
pROKII-ThbZIP1F	CTCTAGAGGATCCCCATGTATCAACCCGTGAGTTCTGGT
pROKII-ThbZIP1R	TCGAGCTCGGTACCCTTAGAACTGAAACATATCAGCGGT
pROKII-ThbZIP2F	CTCTAGAGGATCCCCATGGGGTCTTCGAACTCGAAGCCA
pROKII-ThbZIP2R	TCGAGCTCGGTACCCTCAGAGCCTTCTTCTACCATTTGT
pROKII-ThbZIP4F	CTCTAGAGGATCCCCATGAACCCAAATTACTACTCCAAG
pROKII-ThbZIP4R	TCGAGCTCGGTACCCTTATTGAATCCTATGATTATAGTC
pROKII-ThbZIP7F	CTCTAGAGGATCCCCATGGCGATGCAGACTGGAGTAGGG
pROKII-ThbZIP7R	TCGAGCTCGGTACCCCTACAATACTAAACCCGCTGGCTT
pCAM-C-boxF	AGCTTGACGTCGACGTCGACGTCACCCTTCCTCTATATA AGGAAGTTCATTTCATTTGGAGAGAACACGGC
pCAM-C-boxR	CATGGCCGTGTTCTCTCCAAATGAAATGAACTTCCTTAT ATAGAGGAAGGGTGACGTCGACGTCGACGTCA

Table S3. Primer sequences used in real time RT-PCR.

Gene	GenBank Accession Number	Forward Primers (5'–3')	Reverse Primers (5'–3')
ThbZIP1	FJ752700	AGGATGATCTCCAACCGG	AAGGAATTCAGAGAATTC
ThbZIP2	JX169811	GTTCAGGGATTCATCAGG	ATGGCTATCAATCCAACC
ThbZIP4	JX169812	GATTCGGTGCTTGATGGT	TTGCTCCATGGCCTGCAG
ThbZIP5	JX169813	GTTATCATGCCGGTTCGG	GGTCGTCCGTTCGTGATT
ThbZIP6	JX169814	CTTCTGATGCCCAACTTC	CGATAAAGACCATCATCT
ThbZIP7	JX169815	GAGAGTGTTTCAGATGCT	GTGGTTATATGACTGGAC
Actin	FJ618517	AAACAATGGCTGATGCTG	ACAATACCGTGCTCAATAGG
α-tubulin	FJ618518	CACCCACCGTTGTTCCAG	ACCGTCGTCATCTTCACC
β-tubulin	FJ618519	GGAAGCCATAGAAAGACC	CAACAAATGTGGGATGCT

Table S4. Primer sequences used in construction of the overexpression vectors of ThbZIP1 and ThbZIP1 fused with GFP.

Primer Names	Primers Sequences (5'–3')
GFP-ThbZIP1F	CTCTAGAGGATCCCCATGTATCAACCCGTGAGTTCTGGT
GFP-ThbZIP1R	GAACTGAAACATATCAGCGGTATT
GFP-ThbZIP2F	CTCTAGAGGATCCCCATGGGGTCTTCGAACTCGAAGCCA
GFP-ThbZIP2R	TTACGAGCCTTCTTCTACCATTTGT
GFP-ThbZIP4F	CTCTAGAGGATCCCCATGAACCCAAATTACTACTCCAAG
GFP-ThbZIP4R	TTGAATCCTATGATTATAGTCTAG
GFP-ThbZIP5F	CTCTAGAGGATCCCCATGTTGTCCACAATTCCGGCTAAT
GFP-ThbZIP5R	TGGCCATGCAAAAGACTGCTCATG
GFP-ThbZIP6F	CTCTAGAGGATCCCC ATGGCTTGTATGAAGTTAGGATCT
GFP-ThbZIP6R	CAGGTTTTTCTTGTGCCTTGGATG
GFP-ThbZIP7F	CTCTAGAGGATCCCCATGGCGATGCAGACTGGAGTAGGG
GFP-ThbZIP7R	CAATACTAAACCCGCTGGCTTTAA
GFPR	TCGAGCTCGGTACCCTCACTTGTACAGCTCATCCATGCC

© 2014 by the authors; licensee MDPI, Basel, Switzerland. This article is an open access article distributed under the terms and conditions of the Creative Commons Attribution license (http://creativecommons.org/licenses/by/3.0/).
